# The Relationship between Branched-Chain Amino Acid Related Metabolomic Signature and Insulin Resistance: A Systematic Review

**DOI:** 10.1155/2016/2794591

**Published:** 2016-08-25

**Authors:** Xue Zhao, Qing Han, Yujia Liu, Chenglin Sun, Xiaokun Gang, Guixia Wang

**Affiliations:** ^1^Department of Endocrinology and Metabolism, The First Hospital of Jilin University, Changchun 130021, China; ^2^Hospital of Orthopedics, The Second Hospital of Jilin University, Changchun 130021, China

## Abstract

Recent studies have shown the positive association between increased circulating BCAAs (valine, leucine, and isoleucine) and insulin resistance (IR) in obese or diabetic patients. However, results seem to be controversial in different races, diets, and distinct tissues. Our aims were to evaluate the relationship between BCAA and IR as well as later diabetes risk and explore the phenotypic and genetic factors influencing BCAA level based on available studies. We performed systematic review, searching MEDLINE, EMASE, ClinicalTrials.gov, the Cochrane Library, and Web of Science from inception to March 2016. After selection, 23 studies including 20,091 participants were included. Based on current evidence, we found that BCAA is a useful biomarker for early detection of IR and later diabetic risk. Factors influencing BCAA level can be divided into four parts: race, gender, dietary patterns, and gene variants. These factors might not only contribute to the elevated BCAA level but also show obvious associations with insulin resistance. Genes related to BCAA catabolism might serve as potential targets for the treatment of IR associated metabolic disorders. Moreover, these factors should be controlled properly during study design and data analysis. In the future, more large-scale studies with elaborate design addressing BCAA and IR are required.

## 1. Introduction

Obesity, one of the escalating global health problems, is a major risk factor for the onset and development of diabetes mellitus, metabolic syndrome, cancer, and other chronic disorders [1, 2]. Insulin resistance (IR) is known to play a significant role in obesity-related metabolic disturbances [3, 4]. Several mechanisms are known to be responsible for IR, including carbohydrate metabolism, fat metabolism, and protein metabolism [5]. Despite many years of research, the exact mechanisms between IR and obesity-related metabolic complications are still not fully established. Thus, accurate biomarkers or parameters reflecting insulin resistant state and metabolic risks are essential to understand its mechanism and to prevent obesity-related complications.

Metabolomics is an analytical approach that aims to detect and quantify endogenous small molecule metabolites (<1,500 Da) [6]. Recent studies have suggested that altered metabolites or metabolomics profiles could predict specific metabolic diseases with high accuracy and help to understand related fundamental mechanisms as well as affected metabolic pathways [7, 8]. In addition, metabolomics approach is beneficial to classify personalized “metabolic signature” and make it possible to suggest ideal and individualized therapies effectively [9]. Variable kinds of metabolomics techniques have been applied to generate metabolic profiles from blood, urea, or tissues in human, such as ultraperformance liquid chromatography (UPLC), nuclear magnetic resonance spectroscopy (NMR), and tandem mass spectrometry (MS/MS).

BCAAs are essential amino acids, changing along with the consumption of a protein-containing meal, which include valine, leucine, and isoleucine [10]. Previous studies using metabolomics approaches reported that insulin resistant rats and human had an increased level of circulating BCAA-related metabolites, including valine, leucine, isoleucine, and the sum of the above three amino acids [11–13]. Moreover, some prospective studies found that the plasma concentrations of BCAAs are prognostic for the onset and progress of type 2 diabetes in long-term follow-up [14, 15]. These indicated that BCAA, known as important nutrient signals, might play an important role in the pathogenesis of IR and T2DM [16]. In recent years, several studies have performed many investigations on BCAA and IR as well as diabetes risks across different races, sex, and so on. The results have shown big differences in the BCAA level due to variances in ethnicity, sex, gene expressions, and dietary pattern. However, studies focusing on phenotypic and genetic factors influencing BCAA level are largely lacking. Thus, this systematic review aims to evaluate the relationship between BCAA and insulin resistance as well as later diabetes risks and explore the phenotypic and genetic factors influencing BCAA level and its relationship with IR based on the available studies.

## 2. Method

### 2.1. Literature Search

We searched papers available on MEDLINE, EMASE, ClinicalTrials.gov, the Cochrane Library, and Web of Science for relevant studies from inception to March 2016. A search strategy was applied for MEDLINE according to medical subject headings (MeSH®) terms and some key words. Different possible variations and combinations of the following search terms were used: “metabolomic profiles”, “metabolomic approach”, “serum metabolites”, “metabolic signature”, “branched-chain amino acids”, “BCAA”, “leucine”, “valine”, “isoleucine”, “amino acids metabolism”, “insulin resistance”, “nuclear magnetic spectroscopy,” “NMR”, and “mass spectrometry”. Two persons (Xue Zhao and Qing Han) independently did the search process in order to minimize selection bias. If there were disagreements, a third person will join in and resolve disagreements by consensus (Guixia Wang).

### 2.2. Inclusion and Exclusion Criteria

Inclusion and exclusion criteria were set up before starting paper selection. The following inclusion criteria were applied: (1) all subjects should be adults, which means that age is >18 years and <65 years; (2) participants should be free of any thyroid or metabolic disorders requiring treatment such as diabetes, hypertension, severe dyslipidemia, and coronary heart disease; (3) studies need to include adiposity measures (such as BMI or waist circumference) and HOMA-IR according to the formula glucose (mmol/L) × insulin (pg/mL)/22.5 [17], and serum BCAAs and related metabolites measurement results should be the outcome; (4) metabolomic techniques, such as MS, NMR spectroscopy, and UPLC, were applied to detect metabolite profiles in human blood; (5) principal components analysis (PCA) was used as a means to reduce the complexity of the variables; and (6) only papers published in English were included in our paper.

Studies were excluded if subjects were <18 or >65 years old, or were pregnant, or had diabetes, or had other chronic diseases, or had medication history. The metabolomics profiles extracted from urea are ruled out. Also, reviews, conference abstracts, case reports, and meta-analyses were excluded.

### 2.3. Data Extraction and Analysis

Data on population characteristics and metabolomic profiles were extracted. This procedure was done by two different persons (Xue Zhao and Qing Han). Because of differences in study design, method, and population characteristics, it is not appropriate to perform the quantitative meta-analysis.

BCAAs are known as essential amino acids, including valine, leucine, and isoleucine. Some included studies using BCAA to represent the sum of the above three amino acids. However, other studies point out the specific changes of valine, leucine, or isoleucine. Thus, in this review, we also use BCAA to represent the sum of three amino acids and each amino acid to represent itself in order to sustain the original state of included papers.

### 2.4. Methodological Quality Assessment

To assess the methodological quality of included studies, QUADOMICS tool was applied. QUADOMICS was developed to assess quality issues specific to “-omics” research, including the quality assessment of studies included in systematic reviews [18]. To differentiate high or low quality, studies will be scored from 1 to 16. And studies which scored more than 11/16 are regarded as “high quality,” and studies which scored less than 11 are regarded as “low quality.”

## 3. Results

### 3.1. Literature Search and Study Characteristics

**Figure 1:**
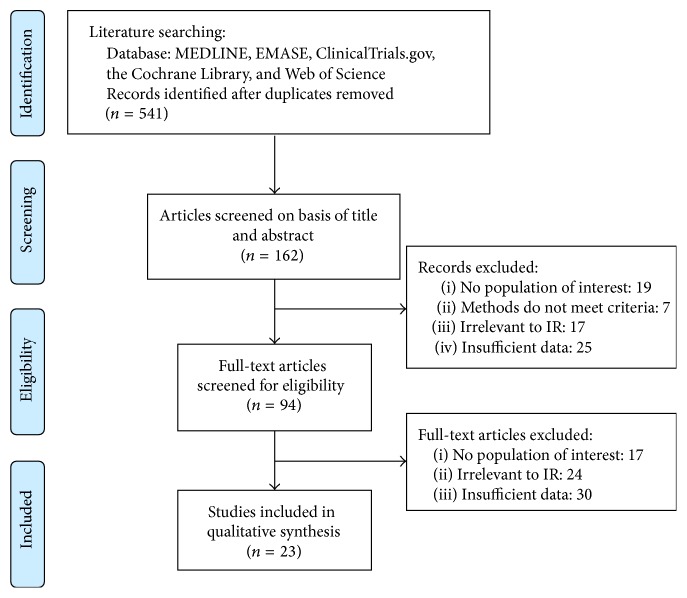
The flow chart of paper selection procedure in this systematic review.

After careful selection, 23 studies (including 20,091 participants) were included in our systematic review, which met the inclusion/exclusion criteria.  Figure 1 presented the procedure of the literature selection based on PRISMA statement [19]. At first, 510 records were identified, and 94 full papers were retrieved. After reading all the papers, twenty-three unique studies providing adequate data were included in this systematic review.

Table 1 showed the details about general population characteristics of the twenty-three studies with 20,091 participants. The included studies were published between 2009 and 2016. Seven studies were conducted in the United States [12, 14, 20–24], four studies were from Finland [25–28], three studies were from China [29–31], two studies were from Canada [32, 33] and Japan [34, 35], and one study was from each of the following countries: UK [36], Korea [37], Singapore [38], Germany [15], and Mexico [39]. The studies varied in sample size from 30 to 7098 with a median of 873. There were seventeen studies enrolling both males and females, and two studies focused on women only [26, 32], while the rest of the four studies address novel findings from males [31, 36–38]. The age of participants in our studies ranged from 18 to 59 years. All studies focused on the serum metabolites and insulin resistance (HOMA-IR). The analytical platforms used for metabolite detection included amino acid analyser, HPLC/fluorescence spectroscopy, ultraperformance LC-MS, GC-MS, FIA-MS/MS, UPLC-Q-TOF MS, NMR spectroscopy, CE-MS, ESI-LC-MS/MS, and UPLC-TQ-MS.

### 3.2. BCAA and Insulin Resistance

Included studies revealed significant alterations of BCAA in obese adults and the intimate association between BCAA and IR. Newgard et al. [12] revealed that obese subjects presented 2.3-fold higher HOMA-IR than lean controls (*P* < 0.0001). Notably, some metabolites showed significant differences between obese and lean controls, which mainly refer to leucine/isoleucine, valine, the aromatic amino acids, and C3 and C5 acylcarnitines (*P* < 0.0001). Furthermore, a significant linear relationship was shown between these BCAA-related metabolites and HOMA-IR (*r* = 0.58, *P* < 0.0001). Consistent results were shown in the study of Würtz et al. [28] on 7,098 young adults, comprising two Finnish cohorts, the Northern Finland Birth Cohort 1966 (NFBC) and the Cardiovascular Risk in Young Finns Study (YFS). They also showed that the BCAA together with other related metabolites was positively associated with HOMA-IR (*P* < 0.0005). Boulet et al. [32] suggested the positive relationship between AA related factors and HOMA-IR (*r* = 0.35, *P* < 0.01). And compared with valine and isoleucine, leucine showed highest correlation with HOMA-IR (*r* = 0.26). Another recent twin study from Bogl et al. [25] consisting of 286 subjects (MZ: 136, DZ: 150) showed HOMA-IR correlated significantly with higher valine, leucine, and aromatic amino acids (AAA) as well as lipid profiles (*r* = 0.30–0.40). In the study by Tai et al. [38] on 263 nonobese Asian Indian and Chinese men, they divided enrolled subjects into low HOMA and high HOMA groups; the results showed individuals with high HOMA presented higher level of valine (*P* = 0.0033) and leucine/isoleucine (*P* = 0.0321) in both Chinese and Asian Indians. Yamada et al. [35] showed the consistent results with previous studies in 94 nonobese Japanese people. Another study [34] on Japanese with normal glucose tolerance also showed positive correlation between HOMA-IR and valine, glutamate, and tyrosine levels, but negative correlation was found in citrulline, glutamine, and glycine levels.

Despite the altered BCAA level in peripheral circulation, BCAA metabolism in target tissue such as subcutaneous adipose tissue (SAT) and visceral adipose tissue (VAT) also showed significant changes in obese and insulin resistant patients [27, 32, 33]. Naukkarinen et al. [27] presented a close correlation between IR and downregulated mitochondrial BCAA catabolism in the adipose tissue of obese cotwins, who showed significantly lower insulin sensitivity and higher plasma insulin concentrations than their lean cotwins.

### 3.3. Elevated BCAAs and Later Risk for Diabetes and Metabolic Disorders

After evaluating all included studies, six prospective studies were found reporting the relationship between elevated BCAAs and later risk of type 2 diabetes [14, 15, 20, 28, 29, 36]. Wang et al.'s study on Framingham cohort [14] showed that 1 SD increment in five target amino acids (isoleucine, leucine, valine, tyrosine, and phenylalanine) was associated with a 57–102% increase in the risk of future diabetes (*P* = 0.0002–0.002). In addition, moderate association was found between baseline amino acids level and HOMA-IR as well as HOMA-beta (*r* = 0.24–0.37, *P* < 0.001). Besides, similar results have been validated in another four large cohorts with long-term follow-up [15, 20, 29, 36]. In Tillin et al.'s study [36] on 801 European and 643 South Asian participants with 19-year follow-up, 227 South Asian men (35%) and 113 European men (14%) developed diabetes [36]. And amino acid metabolites (isoleucine, leucine, valine, phenylalanine, and tyrosine) were associated with incidence of diabetes in both ethnic groups after logistic regression analyses (OR_isoleucine_ = 3.11; OR_leucine_ = 3.36; OR_valine_ = 3.34). In recent studies on Chinese population [29], 51 individuals presented similar level of metabolic markers but higher amino acids level (valine, leucine, isoleucine, tyrosine, and phenylalanine) and 162 controls were enrolled. After 10-year follow-up, the positive correlation was found between the baseline five AAs and future diabetes incidents in these 51 individuals (ORs per SD > 1.5 and *P* <  0.001). However, the baseline metabolic markers failed to predict the risk of diabetes (*P* > 0.05). In the EPIC-Potsdam study with 7-year follow-up, Floegel et al. [15] showed positive correlation between isoleucine, valine, and future risk of T2DM (RR per SD 1.30 [95% CI 1.17–1.43] and 1.27 [1.16–1.40], resp.). Also, the ethnical difference in using BCAA to predict diabetes is presented by Lee et al. [20]; the positive relationship with later diabetes risk was more obvious in Caucasians or in the combined Caucasian and Hispanic group but not in African Americans during 5-year follow-up. In a word, compared with already established metabolic factors, elevated BCAA level can largely improve the accuracy of prediction on future metabolic risk [15].

### 3.4. Factors Influencing BCAA Level and IR

Multiple factors have shown to influence the concentration of BCAA and its correlation with IR. Based on current evidence, these factors can be divided into two main aspects: phenotypic modification and genetic modification. Phenotypic part refers to race, gender, and dietary pattern; and genetic part refers to relative gene variants in BCAA metabolism.

#### 3.4.1. Race Difference in BCAA and Its Relationship with IR

Three studies compared BCAAs outcomes across different races, including Europeans versus South Asians [36], Chinese versus Indian Asians [38], and Caucasians versus African Americans versus Hispanics [20]. The detailed study characteristics about the influence of different race in BCAA level and its relationship with IR were shown in Table 2. Tillin et al. [36] reported that South Asian participants (1,279), compared with European (1,007), had higher serum concentrations of isoleucine (*P* < 0.0001) but weaker correlation with obesity measurements. During 19-year follow-up in this study, higher risk of incident diabetes was also found in South Asian (34%) compared to European subjects (14%). Tai et al. [38] revealed that Chinese individuals with high HOMA had higher levels of amino acids (valine and leucine/isoleucine) than Chinese individuals with low HOMA (*P*_valine_ = 0.005 and *P*_leucine/isoleucine_ = 0.011, resp.). Similar association was also observed in Asian Indians in other amino acids but did not reach statistical significance. The Insulin Resistance Atherosclerosis Study (IRAS) from Lee et al. [20] demonstrated the variances in ethnicity among Caucasians (*n* = 290), Hispanics (*n* = 230), and African Americans (*n* = 165). In stratified analysis by ethnicity, they found negative association between BCAA levels and insulin sensitivity (*S*_I_) in Caucasians and Hispanics [*β*-coefficients: −0.0027 (−0.0035, −0.0020)]. After 5-year follow-up, positive correlation between BCAA and the risk of developing diabetes was only found in Caucasians and Hispanic group but not in African Americans. As for other races (such as Japanese, Chinese, and Finns), similar positive association between BCAA and IR was also presented [29, 30].

#### 3.4.2. Gender Difference in BCAA and Its Relationship with IR

Seven studies addressed gender difference in BCAAs levels [20, 25, 28, 30, 32, 34, 35]. The detailed study characteristics about the comparison of altered BCAA level between male and female were shown in Table 3. The study on 7,098 young adults from Würtz et al. [28] displayed positive association between BCAA (leucine, isoleucine, and valine) and IR, and stronger correlation was found in men compared with women (*P*_leucine_ < 0.001; *P*_isoleucine_ = 0.006; *P*_valine_ = 0.001). In addition, a study from 94 Japanese people [35] revealed that BCAAs (leucine, isoleucine, valine) were positively associated with IR. However, this positive association was only found in women (*r* = 0.354, *P* = 0.016), which did not reach statistical significance in men (*r* = 0.245, *P* = 0.094), while, in a study on 685 participants, Lee et al. [20] found that men had higher level of BCAA as well as BMI, waist-hip ratio, IR, and dietary energy intake compared with women. The gender-dependence was also reported by Xie et al. [30]. They presented that BCAA was correlated with IR only in obese Chinese men; similar results were validated by another two cohorts on Chinese and Americans. Consistent results were also presented by Takashina et al. [34]. Other studies did not show difference in BCAA or IR between male and female.

#### 3.4.3. Dietary Pattern and Weight Loss in BCAA and Their Relationship with IR

Five studies showed the relationship between diet and BCAA [12, 21, 22, 24, 28]. The detailed study characteristics about the influence of dietary pattern and weight loss in BCAA level were shown in Table 4. In the study of Shah et al. [24], BCAAs (valine and leucine/isoleucine) not only correlated positively with baseline HOMA-IR (*r* = 0.50, *P* < 0.0001) but also correlated with 6-month HOMA-IR (*r* = 0.28, *P* < 0.0001) as well as ΔHOMA-IR (*r* = −0.38, *P* < 0.0001) after weight loss. Moreover, during the weight-loss period, valine and leucine/isoleucine presented remarkable reduction (*P* = 0.005, *P* < 0.0001). To test the influence of BCAA intake on plasma BCAA level, they performed baseline and 6-month intake of BCAA in enrolled participants. In Shah et al.'s study, BCAA intake refers to a sum of dietary intake on valine, leucine, and isoleucine intake. The results showed that BCAA intake was weakly related to peripheral BCAA level (*r* = 0.14, *P* = 0.003), but change in BCAA intake was not correlated with plasma BCAA level (*P* = 0.39) and ΔHOMA-IR (*P* = 0.82). The study from Würtz et al. [28] demonstrated that dietary intake, especially for protein intake, was related to fasting metabolites levels (valine, phenylalanine, and tyrosine). However, no relationship was found in protein intake and IR. Batch et al. also presented no difference in glucose metabolism and HOMA-IR between controls and BCAA-supplement group [21]. However, study from Newgard et al. [12] on rats showed positive relationship in BCAA intake and IR, indicating the existence of species difference.

#### 3.4.4. Gene Variants in BCAA and Its Relationship with IR

There were six studies reporting relevant gene variants in BCAA metabolism [22, 27, 28, 32, 33, 39]. The detailed study characteristics about BCAA-related gene variants in specific tissues were shown in Table 5. In study of Badoud et al. [33], plasma valine and isoleucine were positively correlated with HOMA-IR [lean healthy (LH) < metabolic healthy obese (MHO) < metabolic unhealthy obese (MUO)]. To explore the alterations in BCAA-related gene expression, they found downregulations of BCAA catabolism and TCA cycle in subcutaneous adipose tissue (SAT) from MHO and MUO patients compared with LH patients. The key mitochondrial genes involved in BCAA catabolism were significantly influenced, including the increase in* BCAT1* and decrease in* BCKDHA* as well as* BCAT2* in obese group compared with lean group. In two obese groups, MUO group had more significant reduction in* BCAT2* and* BCKDHA* than MHO group. In other studies, Serralde-Zúñiga et al. [39] revealed significant reduction in the gene expressions of* BCAT2* and* BCKDH E1a* in omental adipose tissue of insulin resistant subjects. Similar changes of gene expression were reported by Boulet et al. [32] in visceral adipose tissue (VAT) in obese women with BMI > 30 kg/m^2^ (*P* < 0.05), while, in SAT, only expression of* BCAT2* was decreased. Naukkarinen et al. [27] studied gene variants in weight-discordant monozygotic twins and found that genes related to BCAA catabolism, oxidative phosphorylation, and fatty acid *β* oxidation were downregulated in SAT of obese cotwins compared with their lean counterpart. Due to the impairment in BCAA clearance, higher BCAA level was found in obese cotwins (*P* = 0.016). Another study from Würtz et al. [28] presented the positive correlation between metabolites (isoleucine, alanine, total fatty acids, etc.) and SNP rs1260326 in* GCKR* which was associated with IR (*P* = 0.001). Xu et al. [22] found that individuals carrying C allele of the branched-chain amino acid/aromatic amino acid (BCAA/AAA) ratio-associated variant rs1440581 near* PPM1K* gene may benefit less during weight loss than those without this allele when undertaking an energy-restricted high-fat diet.

### 3.5. Quality Assessment

The study team conducted quality assessment process through QUADOMICS tool [18, 40]. In all the included studies, sixteen studies were defined as high quality, fulfilling more than 11 scores. Two different persons double-checked the general characteristics of included studies (Xue Zhao and Qing Han).

## 4. Discussion

### 4.1. BCAA Metabolism and Its Correlation with IR

Leucine, isoleucine, and valine are known as BCAA. The metabolism of BCAA has been known as a significant factor in the pathophysiology of many multifactorial diseases, such as metabolic syndrome, cancer, and hepatic disease [41]. The catabolism of BCAA contains two important steps. The first step is a reversible transamination reaction converting BCAA to branched-chain a-keto acids (BCKAs). In this procedure, the enzyme branched-chain amino acid aminotransferase (BCAT) played an important role. At the same time, the other partner pair a-ketoglutarate/glutamate takes place with this transamination reaction. The next step in the BCAAs catabolism is irreversible, mediated by the mitochondrial branched-chain keto-acid dehydrogenase complex (BCKDC). This enzymatic step confers oxidation on the BCKAs, leading to the production of NADH, CO_2_, and different end-products [42].

Changes in* BCAT* and* BCKDC* can have significant effects on BCAA catabolism [43, 44]. Because of BCAA dysmetabolism, some potentially toxic intermediates in BCAA catabolic pathway might be accumulated, leading to the impaired cellular or organ function. In some studies, humans or animal models with impaired BCAA metabolism presented higher susceptibility to IR and T2DM compared with controls [41]. Olson et al. [43] showed that high level of toxic BCAA metabolites, rather than BCAAs themselves, could result in mitochondrial dysfunction and apoptosis in *β*-cell.

However, the mechanism between BCAA and IR remains unclear [45]. Studies have shown that persistent high nutrient signaling might lead to the onset of IR mediated by mTORC1 signaling pathway [16, 46]. In recent years, mTOR has been considered as the important link between amino acids and insulin action. Once mTOR has been stimulated, it will activate p70 ribosomal S6 kinase (p70S6K). p70S6K regulates protein synthesis cascade and subsequently leads to the phosphorylation of its downstream target, ribosomal protein S6 kinase (S6K) [44, 47]. Moreover, activation of S6K1 leads to serine/threonine phosphorylation and therefore the inhibition of insulin receptor substrate (IRS)-1. This procedure not only impacts normal insulin signaling but also causes the degradation of IRS-1 [48]. However, much more studies are required in the future to figure out the role of mTOR pathway in elevated BCAA and IR.

### 4.2. Factors Influencing BCAA and Its Relationship with Insulin Resistance

The factors influencing BCAA level and its relationship with IR in this study mainly refer to race, gender, diet, and gene variants. First of all, after generating available studies on different ethnicities, we found that Asian people (Chinese or Japanese) seemed to be more susceptible to higher HOMA-IR and higher BCAA level compared with western people such as Caucasians or Europeans. Although Asian people usually hold lower BMI, studies have shown their higher susceptibility to impaired islet function at the early stages of metabolic disorders [29], while, in studies focusing on western people, Hispanics and Caucasians seemed to have stronger positive association between BCAA and IR than African Americans. Thus, the elevated BCAA level and its relationship with IR seem to be race-dependent. Future studies should extend their sample size in different races in order to validate the above findings. For gender difference, majority of included studies showed obese men had higher BCAA and stronger positive relationship with IR compared with female. This indicated future studies should pay much attention to the gender difference during data analysis and study design. In a word, diverse race difference and gender difference can have significant effects on BCAAs level and insulin resistant state, which should also be validated in studies with larger population.

The influence of dietary pattern on BCAA level and IR was not established yet. For a long time, beneficial effects were presented on the regulation of body weight, muscle protein synthesis, and glucose homeostasis in patients or animals applying BCAA supplementation or BCAA-rich diets [49, 50]. In this study, the results showed that a BCAA-rich diet might have a weakly positive impact in peripheral BCAA level, which was most frequently explained by the insulinotropic properties of amino acids [51] or the decreased glycemic load of high-protein diets. Also, Xu et al.'s study [22] presented a new gene-diet interaction, illustrating that patients with C allele of BCAA/AAA might lose less weight during a weight-loss diet. As for the relationship between BCAA supplement and IR, most studies showed negative results, indicating that the ectogenic BCAA intake constituted an extremely small portion in the onset of IR. However, how BCAAs influence insulin signaling remains unclear. Thus, the relationship between diet, circulating BCAA, and IR deserves further exploration.

Although studying blood metabolites can uncover novel markers of potential clinical relevance, it is difficult to determine the relevant gene variants and specific tissues underlying changes in metabolic pathways. As mentioned,* BCAT* and the* BCKD* complex in BCAA catabolism are broadly expressed in human tissues, indicating that extensive tissues hold the ability of BCAA's transamination and oxidation [13]. Among these tissues, skeletal muscle, adipose tissue, and liver seem to play the central role [45]. In our study, we mainly talked about the gene expressions of BCAA metabolism in adipose tissue based on included studies and revealed the significant role of gene expression changes in BCAA catabolism of adipose tissue regarding pathogenesis of IR. Consistent results were also reported by Pietiläinen et al. [52] and Solini et al. [53]. Another study validating the function of adipose tissue in peripheral BCAA level was performed by Herman et al. [54]. They showed that the transplanted adipose tissue had the capability of catabolizing circulating BCAAs in vivo and indicated the possibility that adipose tissue modulates circulating BCAA levels through regulating BCAA enzymes. All these showed that genetic variants of BCAA catabolism in adipose tissue might have important influences on altered BCAA level and the presence of IR to some extent.

In addition to adipose tissue, several studies have demonstrated the existence of BCAA dysmetabolism in other tissues, while few studies detected relative gene expressions. Shin et al. [55] showed that decreased hepatic* BCKDH* might contribute to the increased plasma BCAAs. Moreover, hypothalamus presented an important role in impaired BCAA metabolism in obesity and diabetes. Their study on rats revealed that short-term overfeeding impaired brain insulin ability to lower BCAAs, which was also associated with reduced* BCKDH* protein in liver. In addition, in a study of 35 subjects, Chevalier et al. [56] revealed the increased postabsorptive protein catabolism with elevated BCAA in obesity was associated with increased gluconeogenesis and led to dysglycemia. In skeletal muscle, Krebs et al. [57] reported that elevated plasma amino acid can induce IR in skeletal muscle through inhibiting the glucose transport/phosphorylation and later glycogen synthesis. As for gut microbiota, the amino acid fermenting bacteria in human gastrointestinal tract present the important role in the digestion and absorption of protein and amino acids [58, 59]. Beyond that, gut bacteria themselves also hold a higher level of BCAA-related amino acids [60]. However, it remains to be known if BCAA from gut bacteria can impact the homeostasis of BCAA in the host and how they influence each other. Future studies should focus on the BCAA catabolic alteration in different tissues and explore the mechanism of altered BCAA and its relationship with IR.

### 4.3. Prospects in BCAA and IR Treatment

BCAAs (valine, leucine, and isoleucine) can be an important and useful biomarker of IR. Besides the reflection of insulin resistant state, BCAA can also give feedback of drug effect. In a randomized, double-blind, controlled study on 25 overweight/obese adults [61], patients using insulin sensitizer therapy showed improved insulin sensitivity and reduced functional metabolites (BCAA) compared to placebo treatment after three months of intervention. Walford et al. [62] found administration of glipizide and metformin can influence the ratio–BCAA/AAAs acutely, and the magnitude of change was dependent on the IR status of participants. The above results demonstrate that BCAA or BCAA/AAAs may be useful biomarkers for monitoring the early response to therapeutic interventions in T2DM patients. Since protein intake or BCAA supplement will not influence plasma BCAA level too much, we highly recommend measurement of BCAA levels in order to capture the whole course of disease progress, including obesity, insulin resistant state, T2DM, and later drug effect. Improvement in this field will definitely deepen our understanding that BCAA (as a biomarker) can reflect not only IR but also pharmacological effects of drug intervention.

### 4.4. Strength and Limitation

Based on current studies, we found that BCAAs could be useful biomarker for IR and predictor for later diabetes risks. To our knowledge, this is the first review focusing on the relationship between BCAA and IR as well as the factors influencing BCAA level. All the eligible studies were published between 2009 and 2016, manifesting the latest development in this field. Although much more studies are largely needed, our study sheds light on the differences in ethnicity, sex, gene expression, and dietary pattern, which are important factors influencing BCAA level that should be controlled and considered when conducting clinical or basic studies. However, several limitations should be addressed here. There were huge heterogeneities in subject and study design; the control group and experimental group were not consistent across studies. This limitation restricts further comparison and data analyzed for meta-analysis.

## 5. Conclusion

In conclusion, this systematic review highlighted BCAAs (valine, leucine, and isoleucine) as useful biomarkers for early detection and diagnosis of IR in nondiabetic patients with obesity and as a valuable predictor for later risk of T2DM. Based on included studies, we found that differences in ethnicity, sex, gene expression, and dietary pattern can influence BCAA level and its relationship with IR. Future studies should focus on the mechanism of BCAA and IR in different tissues and explore the potential treatment targets for obesity and T2DM. Despite the long-time exploration of BCAA and its applications so far, much more studies are still required to bring it to light.

## Figures and Tables

**Table 1:** General population characteristics of included studies in this systematic review.

Study item	Year	Number	Type	Mean age	BMI (kg/m^2^)	HOMA-IR	Detection platform
Chen et al.^a^ [29]	2016	213	Cohort study	FDM: 53.78 ± 1.75 Control: 38.51 ± 0.95	FDM: 25.53 ± 0.50 Control: 24.63 ± 0.27	FDM: 2.13 ± 0.61 Control: 1.67 ± 0.30	UPLC-TQ-MS

Chen et al.^b^ [29]	2016	216	Observational study	52.81 ± 0.71	Obese: 27.26 ± 0.25 Lean: 20.48 ± 0.07	Obese: 2.08 ± 0.54 Lean: 1.11 ± 0.17	UPLC-TQ-MS

Lee et al. [20]	2016	685	Cohort study	54 (47–62)	LT: 26.9 ± 5.0; MT: 28.5 ± 5.6; UT: 29.7 ± 5.4	LT^c^: 2.19 ± 0.39 MT^c^: 1.64 ± 0.6 UT^c^: 1.27 ± 0.25	MS/MS

Takashina et al. [34]	2016	83	Observational study	34.0 ± 5.7	Obese: 26.7 ± 2.3 Nonobese: 21.9 ± 1.8	Obese: 2.1 ± 1.6 Nonobese: 1.1 ± 0.5	HPLC

Bogl et al. [25]	2016	286	Observational study	28.7 ± 0.2	25.4 ± 0.3	1.4 ± 0.1	NMR

Boulet et al. [32]	2016	59	Observational study	47 ± 5.0	Lean: 23.2 ± 1.3 Overweight: 27.0 ± 1.3 Obese: 34.0 ± 3.3	Lean: 1.9 ± 1.4 Overweight: 1.7 ± 0.9 Obese: 2.6 ± 1.0	ESI-LC-MS/MS, ESI-MS/MS

Tillin et al. [36]	2015	2286	Cohort study	European: 52.9 ± 7.3 South Asian 50.6 ± 7	European: 25.65 ± 0.73 South Asian 25.46 ± 0.67	European: 1.71 ± 0.26 South Asian 2.43 ± 0.35	NMR

Yamada et al. [35]	2015	94	Observational study	40.1 ± 9.6	22.7 ± 3.9	1.12 ± 0.72	LC/MC

Liu et al. [31]	2015	30	Observational study	20.5 ± 1.8	Obese: 32.62 ± 2.70 Control: 20.77 ± 1.25	Obese: 1.55 ± 0.12 Control: 1.25 ± 0.11	GC/MS, UPLC-TQ-MS

Badoud et al. [33]	2014	30	Observational study	LH: 51 ± 3 MHO: 50 ± 4 MUO: 48 ± 2	LH: 22.1 ± 0.6 MHO: 30.6 ± 1.1 MUO: 33.0 ± 1.9	LH: 0.65 ± 0.28 MHO: 1.18 ± 0.16 MUO: 2.19 ± 0.42	GC-MS, CE-MS

Xie et al.^a^ [30]	2014	211	Cohort study	Obese: 46.8 ± 6.9 Lean: 45.9 ± 7.2	Obese: 26.7 ± 1.25 Lean: 20.6 ± 0.52	Obese: 1.97 ± 0.83 Lean: 1.11 ± 0.52	UPLC-QTOFMS; GC-TOFMS

Xie et al.^b^ [30]	2014	105	Cohort study	Obese: 47.0 ± 8.0 Lean: 49.9 ± 8.3	Obese: 27.1 ± 1.2 Lean: 18.8 ± 0.7	Obese: 2.20 ± 0.73 Lean: 1.31 ± 0.5	UPLC-QTOFMS; GC-TOFMS

Serralde-Zúñiga et al. [39]	2014	115	Observational study	Non-IR 41 ± 10 IR: 39 ± 11	Non-IR: 29.8 ± 10.9 IR: 44.2 ± 11.8	Non-IR: 1.6 ± 0.6 IR: 5.4 ± 3.2	N/A

Wiklund et al. [26]	2014	78	Observational study	MHO: 39.7 ± 7 MetS: 44 ± 6	MHO: 28.9 ± 3.2 MetS: 30.6 ± 3.4	MHO: 1.6 ± 1.0 MetS: 2.3 ± 0.9	NMR

Batch et al. [21]	2013	1872	Observational study	59.49 ± 11.8	30.05 ± 6.44	2.24 ± 1.87	MS/MS

Floegel et al. [15]	2013	2282	Cohort study	49.5 ± 8.9	26.1 ± 0.09	1.6 ± 0.4	FIA-MS/MS

Xu et al. [22]	2013	734	Cohort study	51.2 ± 9	32.65 ± 3.9	2.36 ± 0.36	N/A

Naukkarinen et al. [27]	2014	32	Observational study	27.4 ± 1.0	Obese: 30.4 ± 1.3 Nonobese: 24.75 ± 1.3	Obese: 2.95 ± 0.5 Nonobese: 1.35 ± 0.3	GC-TOFMS

Cheng et al. [23]	2012	1015	Observational study	56 ± 9	28.3 ± 5.0	2.5 ± 2.2	LC/MS

Würtz et al. [28]	2012	7098	Observational study	31 ± 3	Men: 31.2 ± 2.6 Women: 31.2 ± 2.8	Men: 0.98 ± 0.09 Women: 0.92 ± 0.08	NMR

Shah et al. [24]	2012	500	Cohort study	55.9 ± 8.7	33.9 ± 4.7	2.45 ± 0.5	MS/MS

Wang et al. [14]	2011	2422	Cohort study	Case: 56 ± 9 Con: 57 ± 8	Case: 30.5 ± 5 Control: 30.0 ± 5.5	Case: 3.5 ± 2.6 Control: 3.1 ± 2.3	LC-MS

Kim et al. [37]	2010	60	Observational study	39.55 ± 1.24	Obese: 28.9 ± 0.2 Nonobese: 20.9 ± 0.14	Case: 3.34 ± 0.3 Control: 1.72 ± 0.11	UPLC-Q-TOF MS

Newgard et al. [12]	2009	141	Observational study	51 ± 9	Obese: 36.6 ± 1.33 Lean: 23.2 ± 0.33	Obese: 5.73 ± 0.74 Lean: 2.61 ± 0.22	MS/MS, GC/MS

Tai et al. [38]	2010	263	Observational study	49.25 ± 10.6	Chinese: 24.2 ± 2 India Asian: 24.7 ± 2.1	Chinese: 1.8 ± 0.8 India Asian: 2.63 ± 2	MS/MS, GC/MS

Note: BMI: body mass index; HOMA-IR: homeostasis model of assessment for insulin resistance index; BCAA: branched-chain amino acids; LH: lean healthy; MHO: metabolically healthy obese; MUO: metabolically unhealthy obese; FDM: future diabetes development; LT: lowest third of BCAA; MT: middle third of BCAA; UT: upper third of BCAA; MetS: metabolic syndrome; IR: insulin resistance; non-IR: noninsulin resistance; N/A: not applicable; UPLC-TQ-MS: ultraperformance liquid chromatography triple quadruple mass spectrometry; MS/MS: tandem mass spectrometry; HPLC: ultraperformance liquid chromatography; NMR: nuclear magnetic resonance; ESI-LC-MS/MS: liquid chromatography-electrospray ionization mass spectrometry; ESI-MS/MS: electrospray ionization mass spectrometry; LC/MC: liquid chromatography/mass spectrometry; GC/MS: gas chromatography-mass spectrometry; CE-MS: capillary electrophoresis-mass spectrometry; GC-TOFMS: gas chromatography/time-of-flight mass spectrometry; FIA-MS/MS: flow injection analysis tandem mass spectrometry; ^a^study I; ^b^study II for validation; ^c^data for insulin sensitivity.

**Table 2:** The influence of race in BCAA level and its relationship with insulin resistance.

Study	Country	Race	BCAA-related metabolites identified	Relation between BCAA and IR
Lee et al. [20]	Canada	Caucasians, African Americans, Hispanics	Valine, leucine, isoleucine, total BCAA	Positive, Caucasians plus Hispanics > African Americans

Tillin et al. [36]	Finland	Europeans, South Americans	Isoleucine, leucine, valine, phenylalanine, tyrosine, alanine, glutamine, glycine, histidine	Positive, South Americans > Europeans

Tai et al. [38]	Singapore	Chinese, Asian Indians	Valine, leucine/isoleucine, phenylalanine, tyrosine, glutamate/glutamine, ornithine, alanine, proline	Positive, Chinese > Asian Indians

Note: IR: insulin resistance; BCAA: branched-chain amino acids.

**Table 3:** Comparison of altered BCAA level between male and female.

Study	Number	Men	Female	BCAA and related metabolites identified	Relation between BCAA and IR
Lee et al. [20]	685	45%	55%	Valine, leucine, isoleucine, total BCAA	Positive, male > female

Takashina et al. [34]	83	80%	20%	Valine, isoleucine, leucine, lysine, methionine, phenylalanine, threonine, tryptophan, alanine, arginine, asparagine, *α*-aminobutyric acid, citrulline, cystine, glutamate, glutamine, glycine, ornithine, proline, serine, taurine, tyrosine, histidine	Positive, male > female

Boulet et al. [32]	59	0%	100%	Leucine, histidine, isoleucine, lysine, methionine, phenylalanine, threonine, tryptophan, valine, alanine, arginine, citrulline, cystine, glycine, ornithine, proline, serine, taurine, tyrosine, C3	Positive (leucine)

Yamada et al. [35]	94	51%	49%	Valine, leucine, isoleucine, total BCAA, alanine, tryptophan, phenylalanine, tyrosine, ornithine, glycine, glutamate/glutamine, methionine, lysine, cysteine, aspartic acid, total AA	Positive, male > female

Xie et al.^a^ [30]	211	36%	64%	Valine, isoleucine, leucine, glutamic acid, tryptophan, tyrosine, carnitine, phenylalanine, alanine, beta-tyrosine, creatine	Positive, male > female

Xie et al.^b^ [30]	105	38%	62%	Valine, isoleucine, leucine, glutamic acid, tryptophan, tyrosine, carnitine, phenylalanine, alanine, beta-tyrosine, creatine	Positive, male > female

Würtz et al. [28]	7098	48%	52%	Leucine, isoleucine, valine, glutamine, pyruvate, creatinine, alanine, phenylalanine, phospholipids, fatty acids	Positive, male > female

Note: IR: insulin resistance; BCAA: branched-chain amino acids; AA: amino acids; ^a^study I; ^b^study II for validation.

**Table 4:** The influence of dietary pattern and weight loss in BCAA level.

Study	Diet	Weight loss	BCAA and related metabolites identified	Relationship with BCAA and IR
Batch et al. [21]	BCAA supplement	No	Leucine/isoleucine, valine, tyrosine, methionine, alanine, histidine, phenylalanine; C3; C5	Not related to BCAA and IR

Xu et al. [22]	High & low fat	Yes	Valine, isoleucine, leucine, tyrosine, phenylalanine; tryptophan, BCAA/AAA	Corresponded to gene variants

Würtz et al. [28]	Protein intake	No	Leucine, isoleucine, valine, glutamine, pyruvate, alanine, creatinine, phenylalanine, phospholipids, fatty acids	Positive for valine level Not related to IR

Shah et al. [24]	BCAA intake	Yes	Alanine, leucine/isoleucine, valine, methionine, phenylalanine, tyrosine, glutamate/glutamine, ornithine	Slightly positive for BCAA level Not related to IR

Newgard et al. [12]	BCAA supplement	No	Alanine, valine, leucine/isoleucine, phenylalanine, tyrosine, glutamate/glutamine, aspartate/asparagine	Positive for BCAA and IR (in rats)

Note: IR: insulin resistance; BCAA: branched-chain amino acids; AAA: aromatic amino acids.

**Table 5:** BCAA-related gene variants in specific tissues based on included studies.

Study	BCAA and related metabolites identified	Identified gene variants	Specific tissue
Boulet et al. [32]	Leucine, histidine, isoleucine, lysine, methionine, phenylalanine, threonine, tryptophan, valine, alanine, arginine, citrulline, cystine, glycine, ornithine, proline, serine, taurine, tyrosine, C3	*BCKDHA, BCKDHB, BCAT1, BCAT2, BCKDK *	Visceral adipose tissue, subcutaneous adipose tissue

Badoud et al. [33]	Valine, isoleucine, ornithine, alanine, aspartic acid, glutamine, phenylalanine, methionine, tyrosine, glycine, cysteine, aspartic acid, glutamine/glutamic acid	*BCAT2, BCKDHA, BCKDHB, DBT, *	Subcutaneous adipose tissue

Serralde-Zúñiga et al. [39]	Total BCAA, isoleucine, leucine, valine	*BCAT2, BCKDH E1a, SREBP-1, FTO*	Omental adipose tissue

Xu et al. [22]	Valine, isoleucine, leucine, tyrosine, phenylalanine, tryptophan, BCAA/AAA	*PPM1K (rs 1440581), C allele*	Blood

Naukkarinen et al. [27]	Leucine, isoleucine, valine, alanine, phenylalanine, tyrosine, glutamine, glutamate/glutamine	*Genes of BCAA catabolism (monozygotic twin study)*	Subcutaneous adipose tissue

Würtz et al. [28]	Leucine, isoleucine, valine, glutamine, pyruvate, creatinine, alanine, phenylalanine, phospholipids, fatty acids	*GCKR (rs 1260326)*	Blood

Note: IR: insulin resistance; BCAA: branched-chain amino acids; AAA: aromatic amino acids; *BCKDK*: branched-chain keto acid dehydrogenase kinase; *BCKDHE1a (BCKDHA)*: branched-chain keto acid dehydrogenase E1, alpha polypeptide; *BCKDHB*: branched-chain keto acid dehydrogenase E1 subunit beta; *BCAT1*: branched-chain amino acid transaminase 1; *BCAT2*: branched-chain amino acid transaminase 2; *DBT*: dihydrolipoamide branched-chain transacylase E2; *SREBP-1*: sterol regulatory element binding protein 1; *FTO*: fat mass and obesity associated; *PPM1K*: protein phosphatase, Mg2+/Mn2+ dependent 1K; *GCKR*: glucokinase (hexokinase 4) regulator.
